# Research on Robust and Efficient Optimization Design Methods for Analog Integrated Circuits

**DOI:** 10.3390/mi17020184

**Published:** 2026-01-29

**Authors:** Yunqi Yang, Jiayuan Fang, Huachen Dong, Xiaoran Lai, Dongdong Chen, Di Li, Yintang Yang

**Affiliations:** 1 Faculty of Integrated Circuit, Xidian University, Xi’an 710071, China; yqyang2@stu.xidian.edu.cn (Y.Y.); 25251111564@stu.xidian.edu.cn (J.F.); 23009100660@stu.xidian.edu.cn (H.D.); 23049200331@stu.xidian.edu.cn (X.L.); ytyang@xidian.edu.cn (Y.Y.); 2 The Shaanxi Key Laboratory of Integrated Circuits and System, Xi’an 710071, China

**Keywords:** analog integrated circuits, efficient optimization design, robust optimization design, deep learning technique

## Abstract

With the continuous evolution of CMOS technology towards deep sub-micron and advanced nodes, the challenges of analog integrated circuits (ICs) in performance, design efficiency, and reliability are increasingly prominent. The traditional design methods that rely on manual experience and repeated simulations are no longer able to meet the requirements of complex systems for high performance, high robustness, and fast iteration. In this research, the efficient and robust optimization design methods for analog ICs are systematically reviewed. Firstly, the representative efficient design methods on topology synthesis, parameter optimization, and transfer learning are studied. In addition, with the advancement of technology and the reduction of power supply voltage, parasitic effects and the influence of the external environment on circuits can no longer be ignored. Thus, the robust optimization design methods that consider process, voltage, temperature (PVT), and parasitic effects are further investigated. Then, the advantages and limitations of different methods on design efficiency, performance, and reliability are compared and discussed. Finally, an outlook on the development trend of the efficient and robust design methods for analog ICs is provided, which can provide a reference for subsequent research and engineering applications.

## 1. Introduction

Analog integrated circuits (ICs), as indispensable basic modules in modern electronic systems, are widely used in key fields such as wireless communication, sensor interfaces, power management, and mixed-signal systems [1,2,3,4]. Unlike the highly automated and process-standardized development path of digital circuits, analog IC design has relied on the experience accumulation and iterative iterations of designers, and has limitations of long design cycles, low reusability, and high sensitivity to process, voltage, temperature (PVT) conditions, and operating environments [5,6,7,8]. With the continuous evolution of CMOS technology toward deep submicron and advanced nodes, non-ideal effects have been significantly enhanced, and the impacts of PVT variations, mismatches, and parasitic effects on circuit performance have become increasingly prominent, which has posed severe challenges to the traditional experience-based design methods [9,10,11,12]. How to reduce design complexity, improve design efficiency, and ensure performance robustness while meeting the constraints of multiple performance metrics has become an important issue that urgently needs to be addressed in the field of analog IC design.

Current research on the efficient optimization design methods of analog IC is mainly divided into two aspects: topology synthesis and parameter optimization. For topology synthesis, existing methods have achieved breakthroughs from filling preset modules, to fine-tuning known structures, and further to generating circuit topologies from scratch [13,14,15,16]. However, the above methods still have shortcomings in terms of innovation capability, computational cost, and reliability. In recent years, with the rapid development of optimization algorithms and machine learning technologies, circuit parameter design has gradually been formulated as a high-dimensional, multi-objective, and strongly constrained optimization problem. By introducing intelligent optimization algorithms and performance prediction models, researchers have been able to efficiently explore complex design spaces with limited simulation resources, which can significantly shorten the design cycle and reduce reliance on manual experience [17,18,19,20]. Moreover, the rapid iteration of analog IC processes and numerous topology changes make it difficult for the optimization methods to keep up with design requirements in terms of generalization ability. To further improve design efficiency across technology nodes, transfer learning (TL) methods have been gradually introduced into the automated design of analog ICs. By reusing existing design knowledge, the data requirements and optimization overhead in target scenarios can be reduced [21,22,23]. However, when there are significant changes in process differences and topological structures, transfer models struggle to accurately capture the circuit characteristics and may even introduce negative transfer, leading to designs deviating from the actual feasible solution domain.

The current efficient design methods usually optimize the circuit performance under nominal PVT conditions, which cannot sufficiently consider the non-ideal factors such as different PVT conditions, random mismatches, and post-layout parasitic effects [24,25,26]. In actual manufacturing environments, significant performance degradation may be present under extreme conditions. Therefore, the optimization results usually require extensive manual post-processing and repeated verification, which, to a certain extent, offsets the efficiency advantages brought by early automated design. Consequently, robust optimization design methods have gradually gained attention. Current research on robust design methods mainly focuses on two aspects: PVT variations and post-layout parasitic effects. For different PVT conditions and device mismatches, robust optimization methods ensure that circuits meet performance metrics under different PVT conditions by introducing multi-corner analysis, Monte Carlo simulation, or worst-case constraints [27,28,29]. These methods have clear advantages in improving design reliability, but the optimization process requires repeated high-cost simulations across multiple process corners, leading to a rapid increase in computational complexity. On the other hand, the impact of post-layout parasitic parameters on the performance of analog ICs has become increasingly significant in advanced process nodes, which makes it difficult to ensure the final chip performance only at the schematic level. To address the problem, some studies have begun to incorporate parasitic extraction and post-layout simulation into the optimization loop, which can improve design consistency through parasitic-aware parameter adjustments [30,31,32]. However, layout-level simulation and parasitic extraction are time-consuming. Existing methods often need to make trade-offs between accuracy and computational cost, or rely on approximate parasitic models, which can result in applicability in scenarios with complex layouts. Therefore, how to maintain acceptable optimization efficiency while ensuring parasitic robustness remains a key issue to be resolved in robust optimization design.

**Figure 1:**
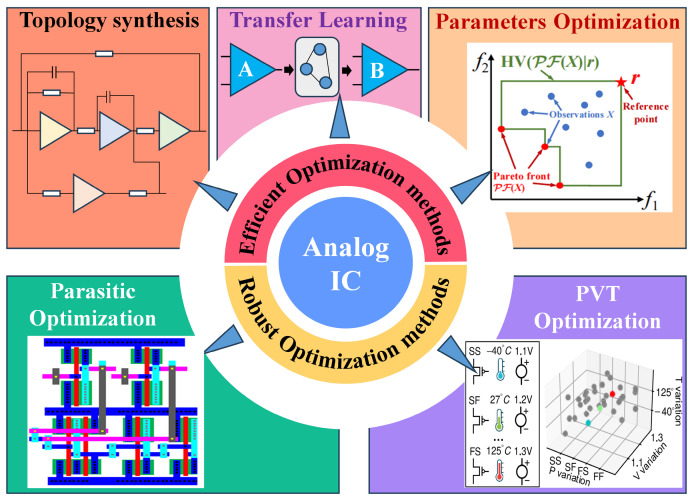
Efficient and robust optimization design methods for analog ICs.

To address the dual challenges of efficient design and robust design, this research conducts a systematic review focusing on efficient and robust optimization design methods for high-performance analog ICs. Firstly, for efficient optimization design, three dimensions are introduced, including: topology synthesis (preset modules-based, known structures-based, generation from scratch), parameter optimization (intelligent optimization algorithm-based, deep learning (DL)-based), and TL methods (different technology nodes, different topology structures). Secondly, for robust optimization design, two sort methods are reviewed, including: robust design considering PVT conditions (reinforcement learning (RL), multi-task learning (MTL)) and robust design considering post-layout parasitic effect (approximate models, layout generators). The core mechanisms for improving circuit robustness and suppressing performance fluctuations are deeply explored. The overall flowchart is shown in Figure 1. This research aims to provide a clear and comprehensive technical overview, identify the advantages and shortcomings of existing methods, and propose future improvement directions, which can promote the evolution of automated analog IC design toward higher efficiency and stronger robustness.

## 2. Efficient Optimization Design Methods for Analog ICs

The design of analog ICs has long relied on the empirical iteration and manual debugging of designers. With the development of technology nodes and the growth of circuit complexity, the efficiency bottleneck of the traditional design has become increasingly prominent. Therefore, it is urgent to propose an efficient optimization design method for high-performance analog ICs. The performance of analog IC design mainly depends on two aspects: topology synthesis and parameter optimization. The quality of the topology directly determines the upper limit of circuit performance, and the parameter optimization needs to achieve the optimal configuration of key parameters such as transistor size and bias current under balancing multi-objective constraints, including gain (Av), unity gain bandwidth (UGB), and power consumption (PC). Meanwhile, transferable methods are required to improve the generality of the approach during technology node migration and topology structure changes. In this chapter, the efficient optimization design methods are introduced from three aspects: efficient topology synthesis methods, efficient parameter optimization methods, and general TL methods.

### 2.1. Efficient Topology Synthesis Methods

Topology synthesis of analog ICs is a core link in automated design, which can directly determine the performance upper limit. However, topology synthesis methods are faced with multiple challenges, including high-dimensional design space, multi-objective constraints, and innovation demands. To solve the above problem, current methods are divided into three categories: topology synthesis methods based on preset modules, topology fine-tuning methods based on known structures, and topology optimization methods of generating from scratch.

#### 2.1.1. Topology Synthesis Method Based on Preset Modules

**Figure 2:**
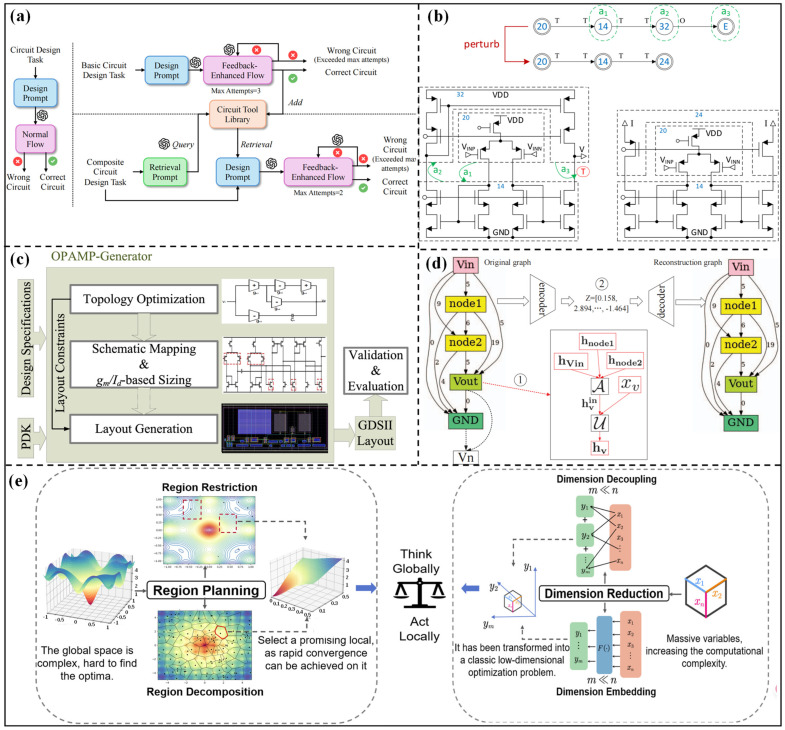
(**a**) Method Overview for AnalogCoder in [34]; (**b**) example of new circuit topology synthesis in [35]; (**c**) overall framework of analog opamp generator in [36]; (**d**) overview framework via graph embedding in [37]; (**e**) region restriction and region decomposition methods in [38].

The core logic of the topology synthesis method based on preset modules relies on the reuse and combination of standardized functional sub-circuits. Through a preset template framework, the design complexity can be decreased. These methods decompose complex circuits into multiple sub-circuit units and achieve the module combination through design rules or optimization algorithms, which can avoid the blindness of designing from scratch. The MOJITO system proposed by Trent McConaghy et al. [16] is a classic representative in this direction. The specific building blocks are adopted as the basic units, and a multi-objective evolutionary algorithm is proposed for topology generation, which greatly improves search efficiency. More than 100,000 operational amplifier topologies are successfully searched by the system, and the performance of the optimized topology is as excellent as those designed by human designers. The complexity of the search space can be reduced through module reuse. However, this method also limits the innovation boundary of the topology structure, making it difficult to generate breakthrough structures beyond the combination of building blocks. Subsequent studies should further expand the application scope of this method. The MOJITO-R tool [33] realizes variation-aware analog circuit structure synthesis through a multi-objective evolutionary algorithm. Finally, 78,643 Pareto-optimal designs are generated, including 982 different topologies, and the performance–topology relationship decision trees can be extracted. With the development of large language models (LLMs), the AnalogCoder topology optimization architecture based on an LLM agent was proposed by Yao Lai et al. [34]. Figure 2a systematically presents the core design framework of AnalogCoder, which deconstructs the full design process of both “basic circuit design” and “composite circuit design”. This method converts circuit design tasks into design prompts to input into the well-trained LLM model, and the automated topology design and error correction can be achieved. Twenty circuits among 24 benchmark tasks without the need for complex model training processes are successfully designed with low deployment costs, which indicates the advantage of the proposed method. However, the adaptability to ultra-large-scale circuits is limited.

A DRL-based circuit topology synthesis framework (CTSF) was proposed by Zhao et al. [35], which takes functional sub-circuits as basic units. This framework consists of six core components: (1) design specifications (gray squares); (2) predefined BB library (PBBL); (3) comprehensive rule set; (4) Policy Gradient Neural Network (PGNN); (5) RL loop (orange square); (6) topology generation and evaluation module. The sub-circuit combination logic is optimized through a signal segmentation mechanism, and existing design knowledge is reused to improve synthesis efficiency. The example of new circuit topology synthesis is shown in Figure 2b. Lu et al. [36] proposed a novel topology synthesis method by decomposing the circuits into several present modules. Figure 2c clearly presents that the transistor sizing is guided by behavioral-level topology, and the layout rules are directly derived from topology constraints, which significantly improves design efficiency. The operational amplifier is decomposed into the input stage, amplification stage, and compensation stage, and the automated optimization design from design specifications to layout can be realized through rule-driven module combination and parameter matching. This method can complete the design without human intervention, which greatly shortens the design cycle. However, the limitations of preset modules make it difficult to generate innovative topologies that break the traditional structures, and the compatibility matching between modules may become a performance bottleneck.

The practical applications for two-stage amplifiers are described in the following. Firstly, the two-stage amplifier is decomposed into several sub-circuit units (such as the input stage, amplification stage, and compensation stage) according to the topology constraints. Then, the MOJITO system, DRL strategy, or AnalogCoder can be adopted to complete the reassembly of submodules and generate new topologies. The quality of each new topology needs to be evaluated by the circuit simulator, and a new two-stage amplifier topology that meets the design specifications can be generated.

In general, the core advantages of the topology synthesis method based on preset modules lie in relying on the reliability of mature circuit modules, which can reduce the design risks and verification costs. Meanwhile, the rule-driven combination mechanism improves design efficiency, making it suitable for standardized and large-scale conventional circuit design. However, the inherent limitations are also obvious. The innovation space is limited by the scope of preset modules, making it difficult to meet the customized needs of niche and high-performance applications. The collaborative optimization between modules is difficult, which can cause problems of performance redundancy or insufficient adaptability.

#### 2.1.2. Topology Fine-Tuning Method Based on Known Structures

The topology fine-tuning method based on known structures explores performance potential through structural optimization and parameter adjustment based on mature topologies, which can reduce the search range of the design space. This method does not need to build the topology from scratch, but accurately captures the mapping relationship between topology and performance through optimization algorithms, which effectively reduces design risks and iteration costs. Žiga Rojec et al. [39] proposed a topology synthesis method based on an evolutionary algorithm. The circuit can be encoded, and the topology structure can be optimized by the non-dominated Sorting Genetic Algorithm (NSGA)-II algorithm. This method not only supports the synthesis of passive, active, and BiCMOS circuits, but also can generate slightly innovative structures based on improving existing topologies. However, the NSGA-II algorithm has a slow convergence speed and is prone to falling into local optima, which makes the design efficiency for high-dimensional complex low.

For the fine-tuning of operational amplifier topologies, a surrogate model was built by Jinyi Shen et al. [40] to quickly evaluate performance and adjust the design parameters. This method effectively improves the constraint satisfaction rate and reduces the number of design iterations. To solve the problem of slow convergence in high-dimensional design space, many scholars have further explored dimension reduction and hierarchical optimization strategies. An operational amplifier topology optimization method based on a variational graph autoencoder (VGAE) was proposed by Lu et al. [37]. The overall flowchart is shown in Figure 2d. From the figure, the topology structure is converted into a directed acyclic graph (DAG) and embedded into a continuous space. Then, the optimal solution in the continuous space is efficiently searched through Bayesian optimization (BO), and the circuit topology is decoded through a graph decoder. The convergence speed is significantly faster than the traditional genetic algorithm, but the topology embedding and restoration process may lead to the loss of some structural information. The multi-layer BO method proposed by Jialin Lu et al. [41] separates topology structure optimization into two layers: the upper layer optimizes the structure of the known operational amplifier topology, and the lower layer optimizes the parameter size. The weights of the two layers are assigned by the weighted expected improvement function. This method can effectively balance the complexity of topology exploration and parameter optimization, but the modeling accuracy and efficiency of the Gaussian Process (GP) model in high-dimensional data scenarios still need to be improved. Lyu et al. [38] reviewed two core topology optimization methods for high-dimensional design space: dimension reduction and region planning, as shown in Figure 2e, where the dimension reduction method utilizes inherent dimensional relationships to guide the optimization process in low-dimensional subspaces. Region planning limits the optimization scope to feasible areas. Based on the above analysis, the ACOB open-source benchmark library is established to provide a systematic algorithm evaluation platform for parameter optimization of known topologies. Meanwhile, the efficient performance prediction model [42] can be trained based on circuit characteristics, which provides accurate initial design points and performance predictions for the size optimization of known topologies.

The practical applications for two-stage amplifiers are described in the following. Firstly, the mature topology of the two-stage amplifier is encoded into a directed acyclic graph DAG. Then, evolutionary algorithms such as NSGA-II and BO are adopted for structural optimization and parameter adjustment. Finally, the optimized topology is restored through a graphic decoder. To balance the exploration complexity and optimization efficiency, a layered optimization strategy, which optimizes topology structure in the upper layer, optimizes parameter size in the lower layer, and allocates weights through expected functions, can be used. The ACOB open-source benchmark library can provide the algorithm evaluation support.

In summary, the core advantage of these methods is to fully use the foundation of existing topologies, which can avoid the blindness of designing from scratch and enable the optimization algorithm to quickly converge to the feasible solution domain. The topology fine-tuning method is particularly suitable for scenarios with high requirements for design stability and performance consistency. However, it is difficult to break through the performance upper limit, because it highly depends on known topologies. When the target scenario has poor adaptability to the original topology, the fine-tuning effect will be greatly reduced. Its application scope is limited by the structural potential and generalization ability of existing topologies.

#### 2.1.3. Topology Optimization Method Generated from Scratch

**Figure 3:**
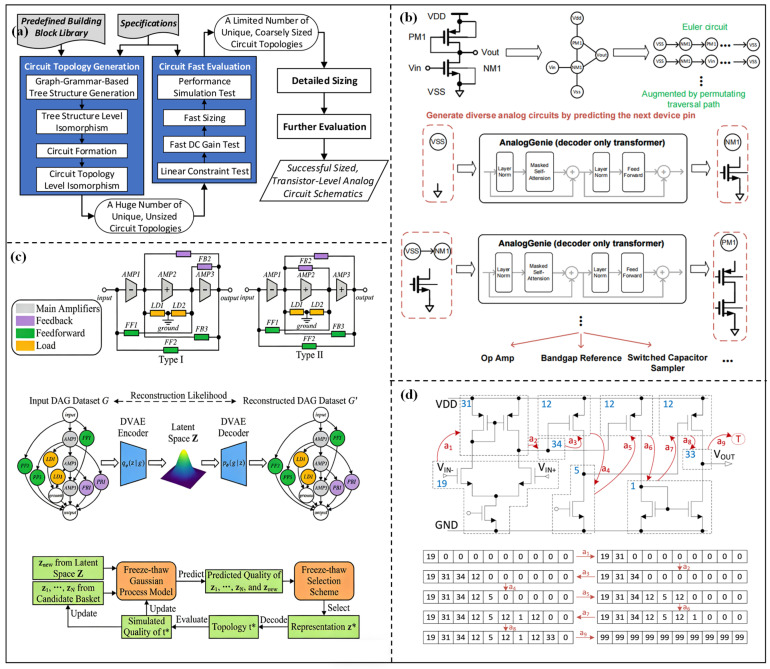
(**a**) Automated topology synthesis framework in [44]; (**b**) analog circuit topology construction diagram by AnalogGenie in [47]; (**c**) automatic topology synthesis framework in [48]; (**d**) circuit topology construction process for three-stage operational amplifier in [50].

The method of generating circuits from scratch aims to break through the limits of traditional topologies, which makes the optimization topology structure not rely on preset templates and provides the possibility to discover high-performance innovative structures. An automatic topology synthesis method for analog circuits based on graph grammar was proposed by Angan Das et al. [43]. The topology structure is automatically generated through the design rules, which are encoded in the form of derivation trees. This method successfully synthesizes operational amplifiers and voltage-controlled oscillators with only thousands of convergent times. Then, the graph grammar framework was further developed by Zhao et al. [44]. The automated topology synthesis framework is shown in Figure 3a. From the figure, the method encodes topology generation as a tree structure. The feasible solutions are quickly screened by symbolic analysis and the gm/ID method, and topology uniqueness is ensured through two-layer isomorphism checking. Three-stage operational amplifiers are successfully synthesized by the framework, and their generation efficiency and reliability are better than those of the existing tool FEATS. However, the tree structure encoding method still limits the complexity of the topology to a certain extent, and its adaptability to large-scale circuits with multi-module interactions is insufficient.

With the development of artificial intelligence technology, DL and RL have become core tools for generating topologies from scratch. Zhao et al. [45] proposed a topology synthesis framework based on deep RL. The building blocks are taken as basic components, and the number of topologies is reduced through hash tables and symbolic analysis. Then, a dedicated RL environment is built to automate topology synthesis, and topology feasibility can be verified through analog simulators. This method can handle large-scale circuits and generate innovative topologies. The reliable schemes can be produced in an average of several hours. However, the design and training process of the RL environment is complex, requiring a lot of computing resources. Chen et al. [46] proposed the TOTAL framework. By simulating the iterative learning process of human designers through RL, the design knowledge can be accumulated through trial and error. Then, the well-trained RL agent can dynamically optimize the topology structure and design parameters. The design efficiency of this framework is higher than that of traditional optimization methods, and it can adapt to the operational amplifier in different application scenarios. However, the generalization and adaptability to non-operational amplifiers still need to be further verified. Gao et al. [47] proposed the AnalogGenie generation engine, and the overall flowchart is shown in Figure 3b. From the figure, AnalogGenie represents each topology as a sequence and generates various analog circuit topologies from scratch by predicting the pin connections of devices. In this engine, a large-scale dataset containing 3350 topologies is built, and a scalable topology description is realized using pin-level graph representation and the Euler tour sequence. This engine can generate 11 types of analog circuits with a maximum of 64 devices, and the proportion of novel topologies is nearly 100%, with better performance metrics than existing methods. However, the construction cost of large-scale datasets is high, and some generated novel topologies may have problems of insufficient stability or practicality. Shen et al. [48] proposed the ATOM framework. The overall framework is shown in Figure 3c, consisting of three core modules: behavior-level topology design space, continuous topological representation learning, and freeze–thaw BO. The design space decomposes the operational amplifier into interpretable functional modules, while the continuous representations of topologies are learned by a variational autoencoder (DVAE). Then, the design parameters are optimized by BO. The success rate and optimization efficiency of this framework are significantly better than those of existing methods. The number of simulations can be reduced by up to 8.15 times, and the generated topologies include both classic structures and innovative schemes. However, the decomposition logic of functional modules still needs to be designed based on domain knowledge. Markus et al. [49] proposed the FEATS exploratory topology synthesis framework to realize comprehensive exploration of the design space through a deterministic circuit generation algorithm and an isomorphism checking mechanism. Unique and feasible topology can be efficiently generated. Zhao et al. [50] proposed an automatic topology synthesis method integrated with a frequency compensation mechanism. The topology construction process from scratch is shown in Figure 3d. At the same time, the compensation structure without preset compensation templates can be synchronously optimized. This method has successfully synthesized multi-stage analog circuits that meet stability metrics. The design efficiency is better than the traditional “topology first, then compensation” method.

This method generates a new topology architecture from scratch, without relying on preset templates, and can balance innovative structure exploration and efficient optimization. Firstly, based on design experience, the two-stage amplifier is decomposed into interpretable functional modules such as the input stage and amplification stage, or encoded into a deduction tree structure through graph syntax rules. By utilizing the deterministic generation algorithm of FEATS and the two-layer isomorphism checking mechanism, topological uniqueness can be ensured to avoid redundancy. Then, topology optimization technologies such as RL and AnalogGenie are utilized to learn the performance of different topologies, and the optimal new topology can be generated. Finally, the evolutionary algorithms can be used to fine-tune design parameters and improve core performance, such as Av, GBW, and PC.

In general, the greatest advantage of this type of method lies in breaking the constraints of traditional topologies, providing an innovative path for the performance breakthrough of analog circuits. These methods are particularly suitable for customized design scenarios that require special performance metrics. However, the challenges cannot be ignored. The high-dimensional design space of the new topology structure leads to high computational costs. Complex check and performance evaluation mechanisms are required for uniqueness verification and feasibility screening. Some generated novel topologies may have problems such as insufficient practicality and high verification difficulty. Thus, the screening mechanism needs further optimization. Table 1 summarizes the optimization circuits, topology synthesis methods, evaluated methods, and optimization results from three aspects of preset modules-based, known structure-based, and generate from scratch methods. From the table, many circuit topologies, such as amplifiers, voltage references, and VCOs, are optimized by current research. The key optimized performance metrics, such as Av, GBW, PM, and PC, are listed to verify the effectiveness of the topology synthesis methods, and the generations and running time are summarized to verify the efficiency. From the results, the method based on preset modules has limited performance improvement due to poor topological flexibility. The method of generating from scratch can discover new circuit structures, but usually requires higher computational costs. Hybrid methods such as PGNN combined with NSGA-II or ATOM exhibit a good balance between performance, novelty, and optimization efficiency.

### 2.2. Efficient Parameter Optimization Methods

Parameter optimization is a key link in the automated design of analog circuits. Due to many mutually coupled performance metrics, the process of achieving optimal parameter design is very complex and highly relies on human experience. Therefore, it is necessary to introduce advanced intelligent optimization algorithms to replace manual design to find optimal parameters. At present, the parameter optimization methods are divided into two aspects: parameter optimization methods based on intelligent optimization algorithms and parameter optimization methods based on DL algorithms.

#### 2.2.1. Parameter Optimization Methods Based on Intelligent Optimization Algorithms

By combining current advanced intelligent optimization methods such as genetic algorithm (GA), particle swarm optimization (PSO), and BO, the optimal parameters can be automatically found in the design space, which has the characteristics of simple implementation and rapid convergence. As a classic tool for multi-objective parameter optimization, GA has been widely used in operational amplifier design. Harsha M.V. et al. [51] proposed the MaxFit GA-SPICE integrated framework. The parameters of two-stage operational amplifiers are successfully optimized through GA, and the optimization performance is excellent with an open-loop DC gain (Av) of 76.31 dB, a unity-gain bandwidth (UGB) of 11.65 MHz, and an area of 262 μm^2^. Purvi Das et al. [52] proposed the MOGA-SPICE framework to improve the optimization effect of the GA through refined objective function design. The optimized area of the two-stage operational amplifier is only 150.55 μm^2^, the optimized UGB reaches 16.78 MHz, and the optimized slew rate (SR) is 15.88 V/μs. Due to the advantages of fast convergence speed and simple implementation, the PSO algorithm performs well in low-dimensional design spaces. Ria Rashid et al. [53] proposed a hybrid PSO algorithm with linearly decreasing inertia weight. A particle generation function and Ngspice survivability test are conducted to screen feasible solutions, and the transistor width and bias current of the two-stage operational amplifier are iteratively optimized. A minimal area of 0.07 μm^2^ under meeting all design constraints can be achieved. In addition, this algorithm is applied to optimize the two-stage operational amplifier under a 65 nm process in [54]. The PSO algorithm is prone to falling into a local extremum. Although the linearly decreasing inertia weight can effectively balance the global and local exploitation capabilities, the search accuracy is still limited in a high-dimensional parameter space. To further improve the search efficiency of optimization algorithms, hybrid optimization and adaptive optimization strategies have become important research directions. A hybrid multi-population optimization algorithm was proposed by Mingyu Li et al. [55], which integrates the search advantages of different populations and balances global exploration and local exploitation capabilities through a complementary mechanism. The optimization efficiency is significantly better than that of a single algorithm, and the design scheme can meet all performance constraints, showing strong engineering practicality. To address the dynamic changes of parameter space during the optimization, a multi-objective optimization method based on adaptive NSGA was proposed by Sridhar et al. [56]. The design space can be efficiently explored, and the optimal configuration of circuit parameters, including transistor size (width/length), bias current, DC input voltage, and compensation capacitor, can be determined.

**Figure 4:**
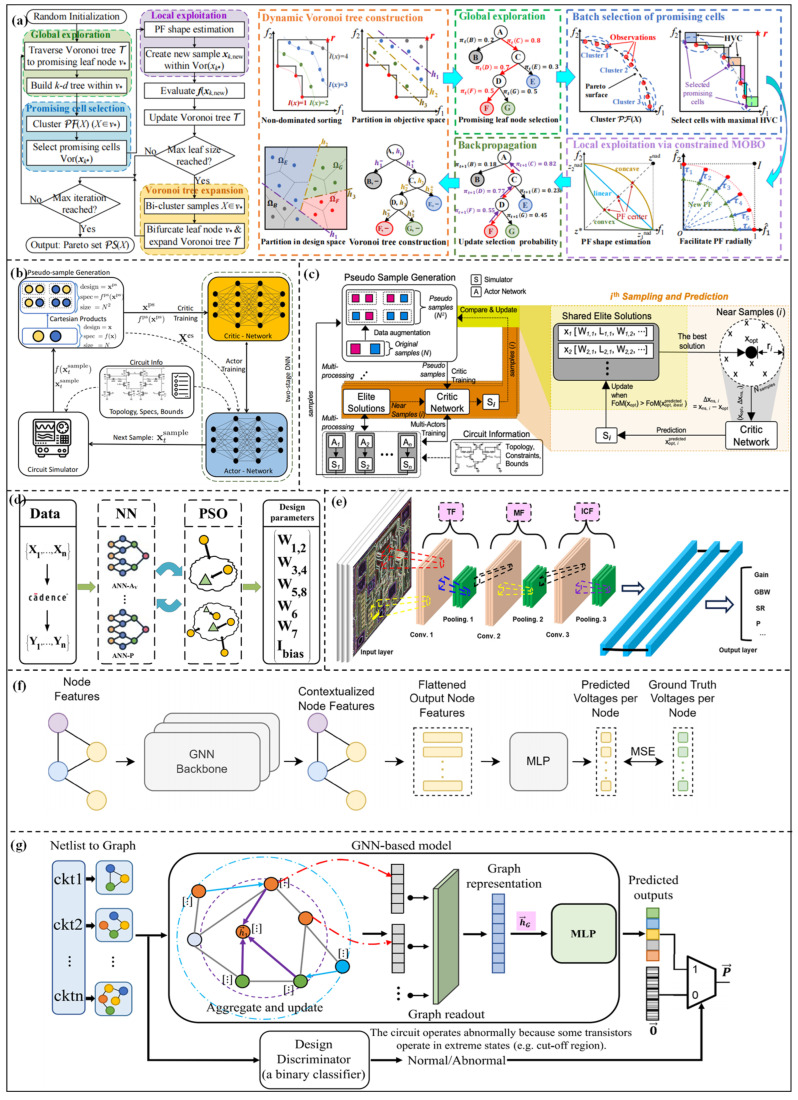
(**a**) Workflow of the proposed VTSMOC method in [58]; (**b**) DNN-Opt framework in [59]; (**c**) MA-opt framework in [60]; (**d**) high-efficiency parameters optimization framework based on neuron network in [61]; (**e**) CNN-IC structure in [62]; (**f**) pretraining model architecture in [63]; (**g**) topology generic DC-model in [64].

Faced with complex parameter optimization scenarios with high dimensions and constraints, BO has become an efficient solution. A derivative-free optimization method for subspaces combined with a batch Bayesian query strategy and GP enhancement was proposed by Gu et al. [57]. By screening dominant regions, the design parameters can be efficiently searched in low-dimensional subspaces without relying on gradient information. The design speed can be accelerated by 2.05–17.65 times, and the running time can be decreased by 1.37–16.11 times for the VCO and charge pump. Zhao et al. [58] proposed a VTSMOC method to optimize multiple competitive black-box objectives. The overall framework is shown in Figure 4a, consisting of four key stages: domain decomposition via dynamic Voronoi tree construction, global exploration via Voronoi tree search, batch selection of promising cells, and local exploitation via constrained multi-objective BO (MOBO). This method achieves high sampling efficiency in high-dimensional constrained multi-objective optimization (CMOO) problems while maintaining low computational costs. Verifications on the charge pump and amplifier show that the sample efficiency and computational efficiency are significantly better than existing methods. Although BO methods currently exhibit good convergence and optimization effects for parameter design, the computational complexity of surrogate models (such as GP, Random Forest) increases with the expansion of circuit scale. Moreover, when the design objectives and constraint conditions are adjusted, the search needs to be reinitialized, making it difficult to adapt to the iterative design process.

The core objective of parameter optimization for a two-stage amplifier is to automatically find the optimal parameters in the design space. Firstly, the design parameters of a two-stage amplifier, such as transistor sizes, bias circuit, compensation capacitor, and the core performance metrics such as Av, GBW, SR, area, and PC, are extracted. Then, a multi-objective optimization function is constructed according to design requirements and constraints. Finally, the design parameters are optimized by intelligent optimization methods such as GA, PSO, and BO. To address the issue of low search efficiency in high-dimensional design space, some mechanisms such as survivability testing, subspace search, and Voronoi tree decomposition are adopted to improve search efficiency. In addition, mixed multi-group strategies or adaptive optimization mechanisms can be integrated into the search process to balance global and local exploitation capabilities.

In general, optimization-based parameter optimization methods can directly optimize the multi-objective function of analog circuits, which do not rely on a large amount of data training, with the advantages of simple implementation and fast convergence speed. However, the search efficiency in high-dimensional parameter space is limited, and some algorithms are prone to falling into local optima. In addition, during the optimization, the circuit simulator needs to be continuously run to verify the performance, resulting in the consumption of a large amount of simulation time and simulation resources in the optimization process.

#### 2.2.2. Parameter Optimization Methods Based on Deep Learning Algorithms

Based on the powerful learning ability of the DL algorithm, the design space of analog ICs can be explored more comprehensively. Compared with traditional intelligent optimization methods such as GA, PSO, and BO, DL can automatically capture the complex correlation between design parameters and performance metrics through massive data training, and even mine circuit features that are difficult to perceive by human experience. It can not only improve the search efficiency in high-dimensional space, but also reduce the consumption of simulation resources. In this section, these methods are mainly divided in two directions: one is to continuously interact with the circuit environment through RL to guide the agent to efficiently explore the design space and avoid falling into local optima; the other is to build a high-precision performance prediction model to replace time-consuming circuit simulation software, which can quickly evaluate the effect of parameter, and greatly shorten the optimization iteration cycle.

RL can effectively find the optimal parameter configuration in the high-dimensional, non-convex, and multi-constrained design space by converting the parameter optimization problem into an interactive decision-making process between the agent and the environment. The DNN-Opt framework proposed by Ahmet et al. [59] adopts an RL-inspired approach based on an actor-critic (deep neural network (DNN)) architecture to realize the size optimization of analog circuits. The overall framework is shown in Figure 4b. First, initial samples are generated from the design space, and pseudo-samples are created to train the critic network for predicting circuit performance. Then, new candidates are produced by the actor network, and the candidates are judged by the critic network. The optimal results can be obtained after the iterations. Verifications on folded cascode operational amplifiers, low dropout (LDO), and other circuits demonstrate that the optimization efficiency can be improved by 5–30 times. Kazuya Yamamoto et al. [65] proposed the GNN-Curio method, which combines the feature extraction ability of a graph neuron network (GNN) with the “curiosity” intrinsic reward mechanism to drive the agent to explore the complex design space. Verifications in circuits with more than 25 components show that the figure of merit (FoM) is improved by more than ten times, and the optimized performance is better than existing deep RL methods. Multi-agent RL has further expanded the boundary of complex circuit parameter optimization. Choi et al. [60] proposed the MA-Opt framework, as shown in Figure 4c. From the figure, the core architecture is the same as DNN-opt. Based on this architecture, parallel training of multiple actors is adopted to share high-quality design schemes in this method. In addition, a shared elite solution set and cooperative near-sampling methods are proposed to improve optimization effects. The proposed method is verified on a two-stage operational amplifier, a three-stage transimpedance amplifier (TIA), and other circuits, and the target metric is improved by 34% compared with DNN-Opt. Meanwhile, Bao et al. [66] proposed the MA-RL framework. Complex analog circuits are decomposed into sub-blocks according to topology. Each sub-block is assigned to an RL agent, and the parameters can be optimized by multi-agent interaction. MA twin delayed deep deterministic policy gradient (TD3) and MA proximal policy optimization (PPO) algorithms are integrated to improve training stability. Verifications on delay locked loop (DLL) and successive approximation register analog to digital converter (SAR ADC) circuits show that the FoM is better than human design and traditional optimization methods. The advantage of the RL-based optimization method lies in the ability to learn design knowledge and strong convergence, but the training process is complex, which requires a lot of computing resources. In addition, the design of the reward function directly affects the optimization effect.

On the other hand, the algorithm needs to continuously adopt the time-consuming circuit-level simulator, which can consume a lot of simulation time and simulation resources for complex circuit modules. DL can also learn the complex mapping relationship between circuit performance metrics and design parameters to quickly and accurately predict circuit performance and shorten the design cycle. Sajadi et al. [67] proposed a transmission gate flip-flop optimization method based on DNN. The key performance metrics, such as dynamic power consumption and setup time, are modeled through DNN using the transistor-level simulation data. The results show that the model is more than 100 times faster than circuit-level simulation. Budak et al. [68] proposed an ESSAB method. By building a dedicated neural network model to predict circuit performance and adopting a new candidate design ranking strategy, the problems of prediction error accumulation and modeling cost under multiple specifications can be solved. Verifications on amplifiers and comparators show that the optimized circuits can meet the strict design specifications, and the convergence speed is significantly improved. Yang et al. [61] built a neural network model to predict the performance metrics of the two-stage Miller-compensated operational amplifier, and the flowchart is shown in Figure 4d. Compared with traditional optimization design methods, the design time can be shortened by more than ten times. Chen et al. [62] proposed a high-accuracy modeling method for analog ICs based on convolutional neural networks (CNN). The model structure is shown in Figure 4e. The design parameters are converted into a two-dimensional sparse matrix according to the circuit topology, and the mapping relationship can be learned through the CNN. The fitting accuracy (R^2^) can reach over 99%.

The circuit topology can be regarded as a network, and each device can be regarded as a node of the network. Therefore, many researchers adopt the graph neural networks (GNN) to learn graph topology, which can improve the modeling accuracy. Kourosh et al. [63] described each circuit as a graph structure, and GNN can be adopted to learn node relationships to predict output node voltages. The model structure is shown in Figure 4f. Studies have shown that predicting output node voltages through a pre-trained GNN model can obtain performance of new topologies, and the sample efficiency is up to 10 times higher than that of randomly initialized models. Wu et al. [69] proposed the Circuit-GNN model. Device connection information can be captured through edge-conditioned convolution. The device group features can be aggregated with the help of Circuit-GIN layers, which can also adapt to different circuit topologies. Verifications on four operational amplifier topologies show that the R^2^ score is improved by 16.7%, which can simultaneously learn the design rules of multiple topologies and break through the adaptation limitation of traditional models to a single topology. Wang et al. [64] proposed a topology generic DC model. The model structure is shown in Figure 4g. The graph structure can be converted into the circuit netlist, and multiple key performances of the amplifier can be predicted through DC parameters based on GNN and attention operations. Verifications on seven amplifiers with different topologies show that the average MAPE is as low as 0.84%, which is 3.88 times faster than SPICE simulation. DL can accurately learn the complex mapping relationship between design parameters and performance metrics. The well-trained models can replace the circuit simulator to achieve efficient and fast performance prediction. However, a large amount of simulation data is required for model training, and the prediction effect on out-of-domain data is very poor. The prediction accuracy of different topology structures and technology nodes is low, and the generalization ability is poor.

The optimization of two-stage amplifiers based on DL methods can be divided into two directions: (1) Firstly, extract the key design parameters and performance metrics of two-stage amplifiers. Construct the actor network to provide the optimization path, and construct the critic network to evaluate the quality of the optimization path. Meanwhile, the corresponding reward function should be set. Then, optimization strategies such as DDPG, PPO, and TD3 are adopted. By continuously interacting with the circuit environment, the design space can be efficiently explored. To improve the search efficiency in the high-dimensional design space of two-stage amplifiers, multi-agent solutions (such as MA Opt and MA-RL) can be adopted. By decomposing two-stage amplifiers into sub-blocks, the agent can be trained in parallel, and the elite solutions are shared. (2) During the optimization, it is necessary to constantly run circuit simulators, which can waste a lot of simulation time. Firstly, circuit data of two-stage amplifiers are previously simulated, and the machine learning models such as DNN, GNN, and CNN (such as ESSAB, Circuit GNN) are established to learn the complex mapping relationship between design parameters and key performance metrics. Thus, the time-consuming circuit simulations can be replaced to quickly evaluate optimization effects and improve optimization efficiency.

DL provides an efficient technical path for the parameter optimization of analog ICs. The complex, high-dimensional nonlinear mapping relationship between design parameters and performance metrics can be well learned from massive data. Through RL, the agent can independently interact with the circuit environment to achieve efficient and global exploration, which can significantly improve the optimization efficiency. The performance prediction model based on DNN greatly shortens the design cycle. However, challenges such as strong data dependence, poor generalization ability across topologies and processes, and complex design of reward functions still exist. In future research, few-shot learning, physical information fusion, and interpretable design need to be further explored to enhance the generalization and reliability of models. Table 2 summarizes the optimization circuits, optimization methods, technology nodes, and optimization results of efficient parameter optimization methods. Many circuits, such as amplifiers, charge pump, VCO, comparator, and LDO circuits under different technology nodes, are optimized. Key performance metrics of optimized circuits are listed to verify the optimization effect. Generations and running time are used to verify the optimization efficiency. The comparison results show that traditional evolutionary algorithms are more robust in performance improvement but require longer running times; the method based on RL or surrogate models can converge in fewer generations, significantly reducing the overall optimization time, but the quality of the results depends on the model accuracy and training strategy.

### 2.3. General Transfer Learning Methods

In the automated design of analog circuits, cross-scenario design (such as technology nodes iteration and topology change) often faces the challenges of data scarcity, high cost of model retraining, and low design efficiency. General TL methods can quickly adapt to the design needs of the target domain (new technology nodes, new topology) by reusing the design knowledge and model parameters of the source domain (existing technology nodes, topology), which can effectively reduce the dependence on target domain data and shorten the design cycle. Thus, TL methods are becoming a key technology to break through the bottleneck of cross-scenario design. This section summarizes the TL methods in two aspects: transfer across technology nodes and transfer between different topology structures.

#### 2.3.1. Transfer Between Different Technology Nodes

The core goal of TL between different technology nodes is to transfer the design knowledge and model parameters of mature technology nodes (source domain) to new technology nodes (target domain). Since the topology structure is not changed during the updating of technology nodes, but only parasitic effects and the electrical properties of the circuit change with the migration of different technology nodes, the well-learned circuit topology features can be reused. The common laws between different technology nodes can be captured by TL to achieve efficient cross-technology nodes learning.

**Figure 5:**
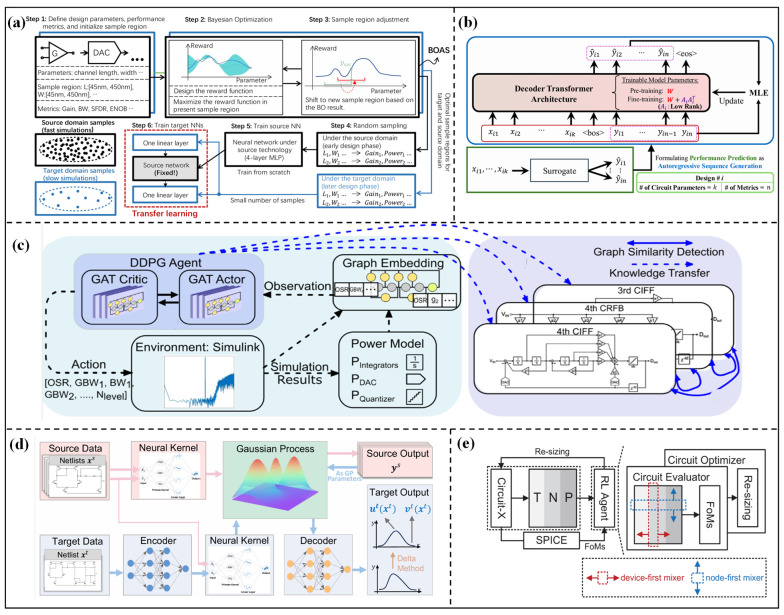
(**a**) Global workflow of the regression model training method via BOAS and TL in [71]; (**b**) INSIGHT Framework in [73]; (**c**) overview framework for the system-level optimization of mixed-signal circuits in [76]; (**d**) knowledge alignment and transfer (KAT)-GP in [74]; (**e**) overview flowchart of Trans-Net in [77].

Wu et al. [70] proposed a transfer strategy of freezing and fine-tuning for the technology nodes migration of operational amplifiers. By freezing the front layers of the model and fine-tuning the back layers, accurate prediction of seven performance metrics can be achieved with only 100 target domain data points. The MAE of the transferred model can be reduced by 50%, and the performance of the transferred model is better than that of the independently trained model based on 1000 target domain data points. For technology migration of full-flow design, a modeling method combining BO-aided sampling (BOAS) and TL was proposed by Liu et al. [71]. The overall diagram is shown in Figure 5a. Firstly, the sample region is initialized, and the region is adjusted via BOAS to filter valid samples. Then, a neural network of the source domain is trained. Finally, knowledge of the source domain is transferred to the target domain through two augmenting linear layers, which can drastically reduce the number of training samples for the target NN. The schematic-level model can be transferred to the post-layout stage and different technology nodes (45 nm/32 nm). Verifications on inverters, amplifiers, and digital-to-analog converter (DAC) show that the dataset for DAC post-layout modeling is reduced by 150 times, and the dataset for amplifier technology migration is reduced by 17 times. The problem of surging data demand caused by technology differences through intelligent sampling has been alleviated. However, the area adjustment strategy of BO sampling relies on prior knowledge of technology differences, and its flexibility is insufficient when adapting to unknown new technologies. Liu et al. [72] realized the full-flow transfer from design specifications to silicon wafers through a neural network surrogate model and the ALIGN layout automation tool. Only a small amount of post-simulation and measured data is needed to improve model accuracy. Verifications on the VCO of the 12 nm FinFET process show that the sizing error of the silicon wafer-level model is reduced to 5%.

Model transfer across multiple technology nodes has become an important development direction. Poddar et al. [73] proposed the INSIGHT framework, as shown in Figure 5b, which formulates analog circuit performance prediction as an autoregressive sequence generation task. Then, the decoder-based Transformer architecture is adopted to predict the performance across 45 nm/90 nm/130 nm multi-technology nodes. When transferring the pre-trained model to a new technology node, the high accuracy performance prediction can be achieved with only a small amount of data fine-tuning. The test R^2^ score is ≥0.95, and the training data are reduced by 60%. The KATO framework proposed by Wei et al. [74], as shown in Figure 5d, first realizes the knowledge transfer in transistor size optimization across 180 nm/40 nm technology nodes. The figure depicts the core architecture consisting of an encoder, a decoder, and a neural kernel (Neuk)-enhanced GP. The encoder maps target domain inputs to the source domain space, and the design knowledge is captured by a pre-trained source GP. The decoder transforms GP outputs to match target domain metrics. The results show that the number of simulations is reduced by 2 times, and the design performance is improved by 1.2 times. This framework also supports cross-topology transfer, but the knowledge alignment mechanism is highly sensitive to technology differences, which needs careful adjustment of the encoding strategy. Wang et al. [75] proposed the GCN-RL method. The circuit topology features extracted by GCN are integrated into the RL agent to achieve the transfer of cross-topology design knowledge. Verifications on two-stage operational amplifiers, LDO, and other circuits show that the optimized FoM is better than that of human experts and BO methods. The performance of optimized circuits is significantly improved after cross-technological node transfer, solving the problem that traditional optimization methods are highly dependent on specific scenarios.

In general, TL between different technology nodes has significantly improved sample efficiency and reduced the design cost through strategies such as freezing and fine-tuning, LoRA, and intelligent sampling. By reusing a large amount of knowledge from mature technology nodes, the demand for target domain data is reduced, and the optimization design after technology iteration can be accelerated. However, there are still many challenges: when the technology node difference is too large (such as traditional processes and advanced FinFET processes), the differences in device physical mechanisms and design rules may lead to negative transfer; some methods rely on prior knowledge, resulting in insufficient generalization.

#### 2.3.2. Transfer Between Different Topology Structures

TL between different topology structures focuses on mining the common electrical characteristics and design rules between different circuit topologies and transferring the design knowledge of mature topologies to new topologies, which can solve the problems of data scarcity and blind exploration of the design space for new topologies. Although different topologies have significant structural differences, there are still commonalities in circuit architecture and electrical characteristics, providing a foundation for knowledge transfer. A graph-guided TL framework for sigma-delta (ΔΣ) ADC was proposed by Wang et al. [76], as shown in Figure 5c. Combining a graph attention network with RL, the electrical similarity between circuits is identified through graph similarity detection, which realizes model transfer among ΔΣ ADCs with different topologies, such as a 3rd-order/4th-order cascade of integrators feed forward (CIFF) and cascade resonator feedback (CRFB). The number of system-level simulations of ΔΣ ADC can be reduced by 1.7–11 times, and the power consumption is improved by 12.4%. TL across multiple functional topologies has become an important breakthrough direction. The KATO framework proposed by Wei et al. [74] is also applicable to different functional topologies through knowledge alignment. Lee et al. [77] proposed the Trans-Net optimizer. The flowchart is shown in Figure 5e, comprising a netlist-mapped circuit matrix, MLP-Mixer modules, a circuit evaluator, and a circuit optimizer. The circuit matrix is generated by a one-to-one mapping from SPICE netlists. The MLP-Mixer fuses device and node information. The circuit evaluator predicts circuit performance, while the circuit optimizer generates transistor sizing. Knowledge transfer across five different topologies, such as operational amplifiers and comparators, is realized. This method can uniformly optimize multiple analog circuits without retraining the model. In specific tasks such as fault diagnosis, multi-topology TL also shows advantages. Gao et al. [78] proposed a graph attention autoencoder combined with a multi-task TL method, which captures the node correlations of three filter circuits. The TL mechanism reuses the node relationships and feature data of the source circuit for fault detection and diagnosis under few-shot conditions. The verification results show that the few-shot detection accuracy reaches 99.51% and the diagnosis accuracy reaches 99.90%.

TL between different topology structures learns the general electrical characteristics through strategies such as graph modeling, unified representation, and selective transfer, realizing cross-topology reuse of design knowledge, which can improve the design efficiency and performance of new topologies. However, there are still obvious limitations: when the topology structure difference is too large, it is difficult to extract common features, and the transfer effect is significantly reduced; there is no unified standard for evaluating topology similarity, which is prone to negative transfer. Most methods are only suitable for specific types of topologies, and generalization to all types of analog circuits still faces challenges.

To comprehensively compare the transfer effect of all methods, Table 3 summarizes the transferred circuits, transfer methods, transfer range, and transfer effect of the present TL methods. Amplifiers, ∆Σ DAC, bandgap reference, TIA, LDO, and CT ∆Σ ADC are transferred. Different transfer strategies (freezing network layers) and hybrid strategies are listed by reusing existing design or model knowledge. In addition, the specific scenarios of TL, including different technology nodes (such as 180 nm → 65 nm, 45 nm → 32 nm), and different topology structures (such as Two TIA ↔ Three TIA, CIFF ↔ CRFB), are summarized. From the results, the TL effect is mainly reflected in three aspects: (1) the improvement of circuit performance, such as the improvement of PC, Av, GBW, and FoM; (2) the improvement of optimization efficiency, including a significant reduction in the number of simulation samples, iteration times, and design time; (3) the feasibility of implementing structural evolution or order expansion in complex systems.

### 2.4. Summary

Centering on the efficient optimization design methods for high-performance analog ICs, this chapter systematically reviews the representative research progress and technical details in three key aspects: topology synthesis, parameter optimization, and cross-scenario TL. In terms of topology synthesis, the method based on preset modules relies on the reliability of mature circuit modules, and can reduce design risks and verification costs, which is suitable for standardized and large-scale design, and has limitations on innovation capabilities. The topology fine-tuning method based on known structures significantly improves convergence speed and stability by reducing the search space, but it is difficult to break through the performance upper limit of existing topologies. The topology optimization method generated from scratch provides the possibility for performance breakthroughs and structural innovation, based on the support of DL and RL, which shows strong exploration capabilities. However, the high computational cost and complex feasibility verification are still the main bottlenecks that restrict the wide application. In terms of parameter optimization, methods based on intelligent optimization algorithms (such as GA, PSO, and BO) have the advantages of simple implementation and strong robustness, which are particularly mature and reliable in medium-dimensional and multi-constraint problems. The parameter optimization methods based on RL and high-precision performance prediction models have significantly improved search efficiency and design upper limits in high-dimensional and strongly coupled design spaces. At the same time, these methods still have significant limitations, such as dependence on large-scale simulation data, insufficient generalization ability across topologies and processes, and complex design of reward functions or model structures. In terms of TL, transfer methods across technology nodes and topology structures have effectively alleviated the problems of data scarcity and repeated training and are key supporting technologies for realizing the large-scale and sustainable evolution of analog circuit design. Strategies such as freezing and fine-tuning, low-rank adaptation, intelligent sampling, and graph representation learning have achieved remarkable results in improving sample efficiency. However, when the process or topology structure difference is too large, the problem of negative transfer is still prominent, and a unified framework has not been formed for the judgment criteria of transfer effectiveness and general representation forms.

In view of the current research of efficient optimization design methods for high-performance analog ICs, this section proposes three directions for future research: (1) deeply integrating device physical mechanism and data-driven methods to improve the interpretability and reliability of models; (2) systematic improvement of few-shot and cross-domain generalization capabilities to reduce the dependence on large-scale simulation data through self-supervised learning, meta-learning, and unified circuit representation; (3) collaborative optimization and closed-loop design of topology-parameter-layout, breaking the existing phased design process and realizing full-process intelligent design from design to tape-out.

## 3. Robust Optimization Design Methodology for High-Performance Analog ICs

Efficient optimization design methodologies continue to mature. However, non-ideal factors encountered by analog ICs in practical manufacturing and application environments—including process variations, device mismatches, and layout parasitic effects—often make optimization results fail to meet the reliability and yield requirements. Therefore, in the design of high-performance analog ICs, introducing robustness constraints to systematically suppress uncertainties has become a critical step. This step enables automated design methodologies to advance from theoretical concepts to real-world engineering practice. The core objective of robust optimization design is to satisfy multiple performance metrics while ensuring that the circuit exhibits stable and consistent performance under varying PVT (process, voltage, temperature) and physical implementation conditions. Thus, recent studies have primarily focused on two aspects: parameters robust optimization considering PVT conditions and post-layout parasitic effects. This chapter systematically reviews robust optimization design methodologies for high-performance analog ICs. Firstly, the robust optimization methods integrated with PVT conditions are summarized and discussed. Then, robust optimization methods addressing post-layout parasitic effects are further reviewed.

### 3.1. Optimization Design Methods Considering PVT Corners

PVT conditions are core factors affecting the performance stability and yield of analog circuits. Fluctuations in circuit performance under different PVT conditions may cause circuits to deviate from design specifications or even fail. Traditional robust optimization methods have limitations, such as high computational cost, low efficiency, and difficulty adapting to high-dimensional parameter spaces. With the development of intelligent optimization technologies, robust optimization methods integrating RL and MTL have gradually become mainstream. By accurately modeling PVT variation laws and efficiently exploring the design space, the optimization efficiency can be significantly improved while ensuring all performance is stable under PVT conditions. Three types of robust optimization methods are discussed in this section, and technical paths, application effects, advantages, and disadvantages are systematically sorted out.

#### 3.1.1. Robust Optimization Based on Reinforcement Learning

**Figure 6:**
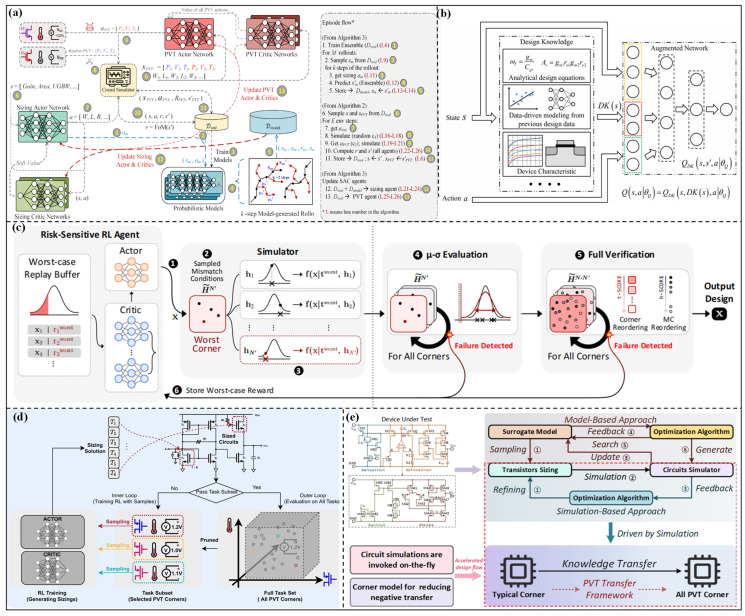
(**a**) Diagram of the AnaCraft duel-play model-based policy optimization in [79]; (**b**) the two-stage critic network with design knowledge in [81]; (**c**) overview framework for GLOVA in [82]; (**d**) RobustAnalog overview in [84]; (**e**) overview of surrogate-based and simulation-based approaches in [85].

Robust optimization methods based on RL take parameter optimization as an interactive decision-making problem, and the PVT conditions are incorporated into the learning process of the RL agent. Based on the feedback from different PVT conditions, the parameter optimization trajectory of the agent balancing performance and robustness is gradually formed through trial-and-error learning. These methods are suitable for handling high-dimensional, strongly nonlinear design spaces with complex constraints without an accurate performance prediction model. Mohsen et al. [79] proposed the AnaCraft framework, as shown in Figure 6a. The figure illustrates the optimization flow, including interactions between actor/critic networks of PVT and transistor sizing, probabilistic models, and the circuit simulator. An adversarial multi-agent RL architecture is adopted, and the relationship between circuit sizing optimization and PVT conditions is defined as a “collaborative-adversarial” problem. Then, the design parameters are optimized through an actor-critic architecture, and extreme PVT conditions are simulated with a circuit simulator to enhance the robustness of the design parameters. A 45 nm CMOS operational amplifier and a 7 nm FinFET data receiver have been optimized. The results show that the number of simulations can be reduced by approximately 3 times, and the runtime can be reduced by about 2 times, while satisfying all PVT constraints. Kim et al. [80] proposed the PPAAS framework for multi-objective and PVT-aware collaborative optimization. By introducing Pareto front target sampling technology, an automatic learning path is constructed. Further optimization is performed using experience replay technology, which not only stabilizes the training process but also accelerates convergence. Validation results indicate that the framework achieves approximately 1.6 times higher sample efficiency and 4.1 times higher simulation efficiency on benchmark circuits, and the multi-performance metrics and PVT robustness requirements are excellently satisfied. Karthik et al. [81] proposed a prioritized RL framework, as shown in Figure 6b, which depicts the two-stage critic network. The first stage embeds the design knowledge (analytical design equations, device characteristics, data-driven modeling) to extract key parameters DK(s). The second stage is an augmented critic network Q_DK_ that integrates the state DK(s) and action for evaluation. High-value training trajectories are prioritized through a non-uniform replay buffer sampling strategy to guide local exploration. A 90 nm CMOS two-stage amplifier is optimized, and the optimized results outperform the Monte Carlo, BO, and standard RL methods. Although knowledge integration reduces blind exploration, the quantification and encoding of design knowledge rely on expert experience, which results in insufficient flexibility when adapting to different circuits.

In addition, trust regions and risk-sensitive analysis have also been introduced into the agent training process to ensure the stability of design parameters. Yang et al. [82] proposed an optimization framework fusing trust region and RL. The parameter optimization is transformed into a constraint optimization problem, and a model-based RL agent is used to implement the trust region strategy. LDO and integrated circuit oscillator (ICO) circuits with TSMC 5/6 nm are optimized; the optimized circuits can achieve a smaller area and have been successfully applied in industrial conditions. To improve the robustness of RL in high-dimensional spaces, risk-sensitive analysis is introduced. The essence of risk-sensitive analysis is to optimize the worst-case scenarios of PVT conditions. Kim et al. [83] proposed the GLOVA framework, as shown in Figure 6c. A risk-sensitive RL algorithm is adopted in this framework, and the performance fluctuations caused by PVT conditions are treated as “risk costs”, which are integrated into the optimization objective function. An actor-critic framework is introduced to optimize design parameters, and a μ-σ evaluation mechanism is used to quantify the performance mean and fluctuation range of different parameter configurations. Combined with a simulation reordering strategy, high-risk parameter combinations are prioritized for verification to reduce invalid computational costs. Validation on 28 nm CMOS strongarm latches shows that this method improves sample efficiency by up to 80.5 times and reduces runtime by 76.0 times, while supporting industrial-grade process corner and Monte Carlo simulation validation.

Except for PVT, the impact of process changes on high-performance analog circuits is also significant. When the process changes, the topology structure of the circuit is not altered, and the electrical characteristics and parasitic effects of the circuit can be changed. Therefore, by learning circuit topology information, optimization algorithms can optimize design parameters under different technology nodes. Many studies have been conducted on robust optimization under process changes. Liu et al. [71] proposed a TL optimization method combined with BO-assisted sampling (BOAS) to reduce the training dataset size required for NN models under different technology nodes. A 17 times reduction can be achieved in the amplifier dataset in the technology nodes migration stage. Xinget al. [74] first proposed a knowledge alignment and transfer optimization (KATO) system to achieve the optimization under process changes. Two-stage and three-stage OpAmps in the 40 nm and 180 nm technology nodes are optimized. Compared with the benchmark scheme, it can achieve up to 2 times the simulation reduction and 1.2 times the design improvement. Wang et al. [75] proposed the GCN-RL method. The GCN model is introduced in actor networks, which can learn circuit topology representations. The circuit performance among five technology nodes can be optimized. The method outperforms other schemes in terms of knowledge transfer ability, achieving better FoM. Lee et al. [77] defined circuit topology by mapping the Spice netlist one-to-one, learned shared information between circuit topologies, and achieved knowledge transfer across technology nodes. The experiment results show that the FoM of 5 circuits under 45, 65, and 180 nm technology nodes is better than the GCN-RL and CAN-RL methods.

The fluctuations in the performance of a two-stage amplifier can be caused by the variation of PVT conditions, and it is necessary to integrate PVT conditions into the optimization process. Firstly, extract multiple performance metrics such as Av, GBW, and PC, and analyze the influence of different PVT conditions. Then, the robust optimization function of the two-stage amplifier is established. Finally, different robust RL strategies such as AnaCraft, PPAAS, and GLOVA are adopted to introduce PVT conditions into the agent learning process, which can achieve efficient and robust optimization meeting all PVT constraints.

Overall, RL-based robust optimization methods improve the stability of design parameters under different PVT conditions through adversarial learning, trust regions, and risk-sensitive analysis. These methods have significant advantages in reducing the number of PVT condition simulations and improving performance consistency under extreme process conditions. However, the training process relies on a large number of interaction samples, and the convergence is sensitive to reward function design, hyperparameter settings, and simulation noise. Thus, such methods still face challenges in computational cost and stability in large-scale industrial applications.

#### 3.1.2. Robust Optimization Based on Multi-Task Learning

Robust optimization methods based on MTL regard parameter optimization under different PVT corners as multiple related tasks. By sharing model parameters or intermediate features, such methods realize the transfer and reuse of design knowledge among different PVT corners. Compared with traditional methods that independently optimize each PVT corner, MTL can significantly reduce redundant computations and improve optimization consistency in complex PVT conditions. Yee et al. [86] provide a general technical support for multi-task robust optimization. Its core is a shared distillation strategy, which uses KL divergence regularization to make the task strategies of each PVT condition close to the global distillation strategy. Wei et al. [84] proposed the RobustAnalog framework, as shown in Figure 6d. The optimization tasks under different PVT conditions are modeled as an MTL problem, and the multi-task deep deterministic policy gradient (DDPG) algorithm is used to train the model parameters. To alleviate gradient conflicts in multi-task training, a k-means task pruning strategy is introduced to eliminate redundant PVT condition tasks, and gradients are normalized through the PCGrad algorithm. Validations on three types of analog circuits show that the simulation costs can be reduced by 14–30 times compared with BO, Evolution Strategy (ES), standard DDPG, and other methods, while achieving a 100% success rate in passing all PVT condition tests. Li et al. [85] proposed the PVT-Transfer framework, as shown in Figure 6e. This framework integrates multi-task optimization and knowledge transfer mechanisms to achieve cross-PVT parameter reuse. High-quality parameter combinations are screened through an assortative mating strategy, and data-driven models are used to capture parameter correlation laws under different PVT conditions. Validations on 180 nm CMOS bandgap and three-output voltage reference circuits show that this method reduces the number of simulations by 60% compared with GCN-RL and improves the figure of merit (FoM) by 10 times compared with manual design. Kong et al. [87] proposed the PVTSizing framework, which integrates the TuRBO algorithm with multi-task RL to construct a three-stage process of “initial sampling—robust optimization—pruning refinement”. High-quality initial parameter spaces are first quickly screened through the TuRBO algorithm. Then, multi-task RL is used to synchronously optimize parameter configurations for different PVT conditions, and invalid computations are reduced by combining critic-aided pruning and index scaling strategies. Four practical circuits are optimized. The sample efficiency is improved by 1.9–8.8 times, and time efficiency is improved by 1.6–9.8 times, while supporting exploration of device mismatch scenarios.

The parameters optimization for a two-stage amplifier in different PVT conditions can be considered as multiple tasks, and the robustness and optimization efficiency are improved through knowledge sharing. Firstly, extract the core performance metrics such as Av, GBW, and PC, and analyze the performance impact under different PVT conditions. Then, an MTL model is constructed to treat each PVT condition as a subtask. By sharing model parameters, the KL divergence regularization distillation strategy is used to achieve the reuse of design knowledge across different PVT conditions. In addition, the multi-task DDPG algorithm can be trained to capture parameter correlations under different PVT conditions. Finally, key parameters such as transistor size and bias current are iteratively optimized to verify the performance consistency under all PVT conditions.

In summary, combined with RL and surrogate models, MTL-based robust optimization methods can alleviate conflicts between different PVT conditions through task grouping, gradient constraints, or selective transfer. These optimization methods perform well under multiple PVT conditions, which can control the overall computational cost and ensure coverage of extreme PVT conditions. However, the performance is highly dependent on task division strategies and condition similarity metrics. When significant differences exist between different PVT conditions, knowledge sharing may lead to negative transfer, which can affect the final design quality.

Table 4 summarizes the optimized circuits, optimization methods, technology nodes, and robust optimization results for the comparison of robust optimization effects when considering PVT conditions. Many circuits, such as amplifiers, LDO, FIA, OSCA, and BGR, are optimized. The optimization effects of each method in robust design are listed, mainly including: (1) the reduction of the required number of simulation samples and simulations, (2) the improvement of simulation and optimization efficiency, (3) the improvement of the overall FoM while meeting all PVT corner validations. The comparison results show that most methods achieve an order of magnitude efficiency improvement while ensuring that all PVT conditions are met, reflecting the significant advantages of intelligent robust optimization in complex circuit design problems.

### 3.2. Optimization Design Methods Considering Post-Layout Parasitic Effect

With the continuous advancement of technology nodes, the impact of post-layout parasitic parameters on analog circuit performance has become increasingly significant. The parasitic parameters include interconnect resistance, capacitance, and device proximity effects. Parameter optimization only at the schematic level often fails to ensure performance consistency in post-layout simulations and even after tape-out. Therefore, incorporating post-layout parasitic effects into the parameter optimization loop to realize collaborative optimization of electrical design and physical implementation has become an important research direction in robust analog circuit design. Existing robust optimization methods considering post-layout parasitic effects can be mainly divided into two categories: parasitic-aware optimization methods based on approximate parasitic modeling and parasitic-aware optimization methods based on automatic layout.

#### 3.2.1. Parasitic-Aware Optimization Methods Based on Approximate Parasitic Modeling

**Figure 7:**
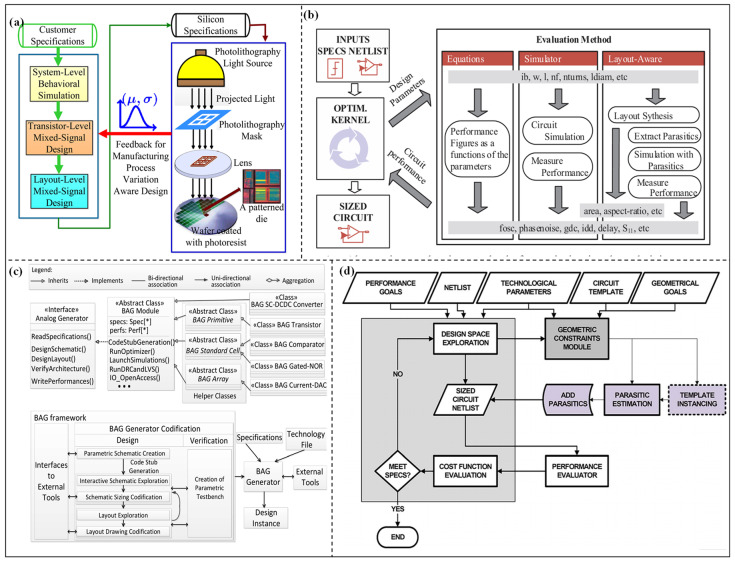
(**a**) Incorporating manufacturing process variation in [90]; (**b**) alternative evaluation methods for optimization-based sizing in [93]; (**c**) implement diagram with the BAG in [97]; (**d**) block diagram of the layout-aware sizing methodology in [99].

Approximate model-based methods construct simplified parasitic estimation models to quickly evaluate the impact of parasitic effects on circuit performance, which do not need to complete layout generation and parasitic extraction. Efficiency and physical realization can be balanced in the parameter optimization process. Oleg et al. [88] proposed a metamodel-driven fast simulation method for nanometer CMOS circuits, which is focused on layout parasitic awareness. Parasitic extraction results are characterized by constructing polynomial metamodels, and the simulation efficiency and accuracy can be improved. Ghai et al. [89] proposed an RFIC design flow. Taking a 90 nm CMOS VCO as an example, the impact of parasitic effects is analyzed through parameterized parasitic netlists, and design parameters are adjusted by combining conjugate gradient optimization. Only one physical design iteration is required to reduce the deviation between the oscillation frequency and the target from 43.5% to 4.5%, which successfully meets the 2 GHz frequency requirement. This method has strong targeting and high optimization accuracy, but the construction of parameterized netlists relies on domain knowledge, which results in poor flexibility under different circuits. Saraju et al. [90] extended this idea by integrating process variations and parasitic effects and used PSO to optimize transistor size and oxide layer thickness. Figure 7a illustrates the core logic of incorporating manufacturing process variations into design to enhance chip yield. The probability density function (PDF) models are established to provide a foundation for subsequent statistical estimation and circuit optimization. Under worst-case process variations, this method achieves a 25% power reduction and controls the center frequency deviation within 1%.

Constraint-driven and symbolic modeling further enhance the practicality of approximate models. Husni [91] proposed a constraint-driven sizing optimization method considering layout parasitic effects. The method enumerates device layout possibilities, extracts parasitic parameters using a field solver, and then iteratively optimizes using a deterministic nonlinear optimization algorithm. Validation on operational amplifiers shows that the deviation between post-layout simulation and schematic simulation is significantly reduced, which avoids the performance failure problem of traditional sizing optimization. However, the complexity of device layout enumeration grows exponentially with the increase in circuit scale, making the method only suitable for small- and medium-sized circuits. Tseng et al. [92] proposed the symbolic extraction paradigm. Parameterized layouts are described through the GBLD language, and mathematical equations of parasitic parameters in symbolic form are extracted. This method supports simple symbolic extraction and conditional symbolic extraction, and does not require repeated layout generation, which can quickly calculate accurate parasitic values, and significantly shortens the design cycle. However, the difficulty of deriving symbolic equations increases with the complexity of the layout, making the method difficult to adapt to non-parameterized complex layouts. Nuno et al. [93] proposed a layout-aware sizing optimization method. Figure 7b demonstrates a comparison diagram of different optimization-based analog IC sizing methods. The figure distinguishes different performance evaluation paths, which can output the optimal parameters that meet geometric constraints and parasitic requirements, and intuitively demonstrates the integration logic of layout information in sizing. Parasitic effects are estimated through floorplan and global routing, and design parameters are optimized by the NSGA-II algorithm. Validations on two-stage operational amplifiers and folded cascode operational amplifiers with UMC 130 nm process show that the parasitic estimation accuracy is close to that of commercial tools, and the optimization time is significantly shortened. However, the parasitic estimation relies on the accuracy of the floorplan.

Machine learning has strong learning capabilities and can learn complex nonlinear mapping relationships between circuit topology, design parameters, parasitic parameters, and performance metrics. Compared with the mechanistic models, machine learning models can provide more accurate parasitic predictions, achieving dual improvements in efficiency and accuracy. Samuel et al. [94] proposed a multi-objective BO method resistant to input noise. With multivariate value-at-risk (MVAR) as the core indicator, the method models parasitic parameters by a GP surrogate model and uses BO for robust optimization of design parameters. This method is suitable for multi-constraint optimization, but the modeling efficiency of the GP model in high-dimensional parasitic parameter scenarios needs to be improved. GNNs have become the mainstream model for parasitic prediction due to their ability to effectively capture circuit topology information. The graph optimization framework proposed by Ren et al. [95] converts circuit schematics into heterogeneous graphs and predicts parasitic capacitances and device parameters (such as sizing parameters and LDE parameters) based on the GNN model. The average prediction R^2^ reaches 0.772, which is 110% higher than XGBoost, and the simulation error estimated by designers is reduced from over 100% to 10%. The parasitic effect can be predicted only through topology information without layout details. However, the method is sensitive to the construction logic of heterogeneous graphs, and the completeness of topological representation affects prediction accuracy. Oleg et al. [96] proposed a metamodel-assisted robust optimization design method that trains a neural network model to predict parasitic parameters in circuits and optimizes PLL performance by combining the proposed bee colony algorithm. The optimized results achieve optimal designs for two different wireless specifications, while demonstrating flexible and robust characteristics. Compared with swarm-based optimization algorithms, this method runs 2.4 times faster on the same metamodel.

Firstly, a parasitic approximation model based on domain knowledge can be constructed through polynomial meta models or parameterized layouts described in GBLD language. Then, based on input noise robustness requirements, intelligent optimization algorithms such as BO, PSO, and NSGA-II can be used to iteratively optimize key design parameters such as transistor size and oxide thickness, and reduce the deviation between optimized performance and actual performance. The parasitic estimation accuracy is close to that of commercial tools, which can significantly shorten the design cycle and achieve efficient and robust optimization of the two-stage amplifier.

Overall, parameter optimization methods based on approximate parasitic modeling aim to reduce the computational complexity of post-layout parasitic analysis and seek a balance between efficiency and accuracy through different levels of parasitic modeling methods. Mechanism-based approximate model methods explicitly embed complex parasitic effects into the parameter optimization process through polynomial metamodels, parameterized parasitic netlists, symbolic extraction, or layout-level estimation. Machine learning-based modeling methods learn implicit mapping relationships between circuit topology, design parameters, and parasitic effects, which have higher accuracy of parasitic prediction. The design parameters can be robustly optimized through BO and evolutionary algorithms. However, the accuracy of such methods is highly dependent on data, making them difficult to meet the design requirements of large-scale, non-parameterized layouts. In future research, the integration of mechanisms and data-driven models is expected to further improve the efficiency and reliability of post-layout optimization for analog ICs.

#### 3.2.2. Parasitic-Aware Optimization Methods Based on Automatic Layout

Layout generator-based methods deeply integrate the process of parameter optimization, automatic layout generation, and parasitic extraction. A closed-loop process of “parameter optimization—layout generation—parasitic feedback” can be constructed, and the disconnect between electrical design and physical implementation is fundamentally alleviated. Such methods can provide high-precision parasitic information, significantly improving the consistency and reliability of the final design. The Berkeley analog generator (BAG) framework proposed by Crossley et al. [97] is a foundational work in this direction, and the optimization process is shown in Figure 7c. The BAG can implement the entire design flow from specifications to layout, which consists of two parts: (a) a UML class diagram of the BAG framework; (b) a demonstration of the design flow, covering parametric schematic creation, layout exploration, and generator coding. The full-flow design of VCOs and switched-capacitor regulators with a 65 nm process is successfully designed by the framework, which demonstrates great potential for electronic design automation. Kourosh et al. [98] proposed a BagNet optimization framework to robustly optimize analog circuits based on evolutionary algorithms, DNN, and BAG. This method only requires 435 simulations to meet all design specifications. Rafael et al. [99] proposed a method integrating parasitic-aware sizing optimization and geometric constraint sizing optimization, as shown in Figure 7d. The flowchart takes performance goals, netlists, and geometric requirements as inputs and forms an iterative closed-loop through design space exploration, geometric module optimization, parasitic estimation, and performance evaluation. Through templated layout generation technology, the method extracts parasitic parameters by analytical methods and layout sampling. Geometric objectives such as area and aspect ratio are considered in the optimization. The optimized operational amplifier design meets all electrical and geometric constraints, which can significantly reduce iterations between electrical and physical design.

The Opamp-Generator framework proposed by Lu et al. [36] further realizes full-flow automation design from specifications to GDSII layout. A VGAE model is used to optimize the topology structure, and the transistor sizing optimization is completed through the gm/Id method. Layouts are automatically generated, and parasitic parameters are extracted by the Magical tool. The generated three-stage operational amplifier has excellent performance under a 180 nm process. Santeri et al. [100] proposed an efficient, fully programmatic, and automated post-layout simulation optimization method for analog circuit design. Firstly, the design problem is formulated as a nested function, and the problem is split into both electrical and physical domains while selecting the optimization starting point. Secondly, the circuit can be optimized by backpropagation technology. Finally, layout parasitic effects can be extracted to adopt the post-layout simulation.

The optimization loop of “parameter optimization layout generation parasitic feedback” for a two-stage amplifier can achieve the combination of preliminary design and physical implementation. Firstly, based on the design specifications of a two-stage amplifier, preliminary optimization of transistor parameters is completed through the gm/Id method or evolutionary algorithm. Then, the layout of a two-stage amplifier can be automatically generated by the BAG optimizer, and geometric target constraints such as area and aspect ratio are extracted. Through analytical methods, layout sampling, or magical tools, parasitic parameters can be accurately extracted and fed back to the parameter optimization stage in real time. Key parameters such as transistor size and bias current can be iteratively adjusted to solve the problem of disconnection between early design and physical implementation. Finally, the optimal solution can be found by a small number of simulations, which satisfy all electrical specifications and geometric constraints.

Parasitic-aware optimization methods based on automatic layout achieve accurate extraction and real-time feedback of parasitic parameters through an integrated flow, which can significantly reduce the design cycle and improve design robustness. The core advantage lies in high parasitic estimation accuracy, which can meet both electrical performance and physical constraints. However, the development and maintenance costs of layout generators are high, and such methods are highly dependent on specific circuit types and layout templates. The automatic layout and parasitic extraction process of complex circuits may also bring additional computational overhead, limiting the efficiency advantages in large-scale design space exploration.

### 3.3. Summary

This chapter focuses on robust optimization design methods for high-performance analog ICs. The representative research progress and technical context are systematically reviewed in two key aspects: PVT-aware parameter optimization and post-layout parasitic-aware optimization. In terms of parameter robust optimization considering PVT conditions, RL-based methods have shown strong adaptability in high-dimensional, strongly nonlinear design spaces through interactive learning with multi-PVT conditions, but the training stability and simulation cost remain restrictive factors. MTL-based methods effectively reduce redundant computations and improve sample utilization efficiency by sharing design knowledge among different PVT conditions, but are prone to negative transfer problems when PVT differences are significant.

In terms of parameter robust optimization considering post-layout parasitic effect, approximate parasitic models achieve fast optimization without complete physical implementation and are suitable for rapid exploration and feasibility verification in the early design stage, but the accuracy is limited by model precision. By deeply coupling parameter optimization, layout generation, and parasitic extraction, parasitic-aware optimization methods based on automatic layout can meet both electrical and physical constraints, which can significantly improve post-layout consistency, and are suitable for full-flow automated design, but are highly dependent on layout templates and circuit types, with high development and computational costs.

Overall, robust optimization design methods effectively make up for the shortcomings of previous optimization design methods in engineering reliability by explicitly modeling uncertain factors. However, current research still needs to consider the following questions: the trade-off between robustness constraints and computational efficiency in high-dimensional parameter spaces, the lack of a unified modeling and evaluation framework among different robust optimization strategies, and the integration of the design flow from schematic to layout. Such issues can provide directions for subsequent research on automated robust analog circuit design methods.

## 4. Conclusions and Perspectives

In this research, the recent studies focused on the efficient and robust optimization design of analog ICs are systematically reviewed and comprehensively analyzed. The efficient design methods, including topology synthesis, parameter optimization, TL, and robust design methods considering PVT conditions and parasitic effects, are investigated respectively.

Section 2 focuses on summarizing the efficient design methods, such as topology synthesis, parameter optimization, and TL. For topology synthesis methods, preset module-based and known structure-based topology synthesis methods rely on mature circuit structures, which have the advantages of low design risk and low verification cost, and are suitable for standardized and large-scale design requirements. However, the search space is limited, which can make it difficult to achieve performance breakthroughs at the structural level. The topology optimization method generated from scratch has shown stronger exploration ability under the promotion of DL and RL algorithms, which can provide possibilities for performance improvement and structural innovation. However, the high computational cost and complex feasibility verification still limit the applications in engineering. For parameter optimization methods, traditional intelligent optimization algorithms perform stably and reliably in medium-dimensional and multi-constraint problems. The DL-based methods significantly improve search efficiency in high-dimensional spaces, but require higher demands on simulation data and generalization ability. Thus, TL methods are proposed, which are of great significance in alleviating data scarcity and reducing the cost of repetitive training. However, when there are significant differences in technology nodes or topology, the problem of negative transfer may arise.

Section 3 systematically analyzes the robust optimization methods considering PVT conditions and parasitic effects. For the robust design considering PVT conditions, RL and multi-task learning methods can balance the performance constraints of multiple PVT conditions, which can improve the design reliability. However, the computational cost, strategy stability, and unified evaluation criteria still need to be improved. For the robust design considering parasitic effects, the approximate parasitic modeling methods based on mechanism models and ML models have low implementation cost and strong flexibility, which is suitable for rapid exploration in the early stages of design, but the accuracy is limited by the modeling accuracy. The automatic layout method can simultaneously satisfy electrical and physical constraints, with high consistency in post-layout, but the development costs are high. Overall, robust optimization methods can effectively compensate for the shortcomings of efficient design methods in engineering reliability, and are an important support for promoting the practical application of analog IC automation design.

Although significant progress has been made in efficient and robust optimization design methods of analog ICs, there is still a certain gap between large-scale, universal, and full process intelligent design of analog ICs. Based on the analysis of the above methods, future research can be focused on the following three directions.

### 4.1. Optimization Design Methods from Black Box Optimization to Physical Perception

The existing optimization design methods for analog ICs mostly consider the circuit as a black box system, and the optimization process mainly relies on simulation data and numerical feedback, which have the limitation on interpretability and engineering reliability. In future research, the optimization design methods need to integrate device physical models, circuit mechanisms, and data-driven methods to guide models to search while complying with the inherent laws of circuits, which can enhance the interpretability and verifiability of design results while improving optimization efficiency and providing more reliable support for engineering applications. 

### 4.2. Improvement of Model Generalization Ability for Different Applications

The current intelligent optimization methods based on DL algorithms generally rely on numerous simulation data, which have poor generalization ability in new technology nodes and new topology structures, resulting in high design costs. In future research, self-supervised learning, meta learning, and a unified circuit structure representation can be studied to explore the common features under different application environments, which can enhance the applicability and stability of the model in new technology nodes and new design scenarios.

### 4.3. Full Process Optimization Design from Front-End to Post Simulation

The existing optimization design methods for analog ICs often adopt a staged approach, with insufficient coupling between topology synthesis, parameter optimization, and layout, which can easily lead to performance degradation and multiple iterative designs. In future research, collaborative optimization from front-end to post-simulation needs to be investigated. By constructing a closed-loop design framework driven by parasitic awareness, the optimization process, integrating electrical design and physical implementation, can be achieved, which can gradually move towards intelligent design of the entire process from design specifications to chip implementation.

## Figures and Tables

**Table 1:** Summary of the efficient topology synthesis methods.

Methods	Refs.	Circuit	Topology Synthesis Methods	Evaluated Methods	Optimization Results
Preset modules-based	[34]	Common source amplifier, three-stage amplifier, folded Cascode amplifier	AnalogCoder	PySpice	Common source amplifier: Av = 13.98 dB; Three-stage amplifier: Av = 44.13 dB; Folded Cascode amplifier: Av = 13.98 dB
Known structure-based	[39]	Voltage reference	NSGA-II	NgSpice	PC = 1.8 μW, PSRR = 89 dB, generations = 1610, time ≈ 35 h
	[41]	Three Novel amplifiers with compensation	Bi-level BO	Cadence	**Run1:** Av = 90.34 dB, PM = 56°, GBW = 3.3 MHz, PC = 69.44 μW; **Run2:** Av = 90.5 dB, PM = 47°, GBW = 3.1 MHz, PC = 102.6 μW; **Run3**: Av = 87.2 dB, PM = 45.4°, GBW = 3.4 MHz, PC = 91.7 μW; Generations ≈ 8000; Running time ≈ 9 h
Generate from scratch	[43]	Novel amplifier, voltage Controlled Oscillator (VCO)	Graph Grammar Based Approach	HSpice	**Novel amplifier:** Av = 42.15 dB, UGB = 9.2 GHz, PM = 56°, generation = 100; **VCO:** Oscillation frequency = 4.17 GHz, tuning range = 0.12 GHz, generate = 50
	[44]	Novel multistage amplifiers	GCTG	Cadence	Av = 70.31 dB, BW = 66.48 MHz, generate 388 tree structures within 0.06 s
	[45]	High-Av amplifiers, high-BW amplifiers, novel amplifiers	PGNN + NSGA-II	Cadence	**High-gain Opamps:** Av > 100 dB, PM = 60°, UGB = 10 MHz; **High-BW Opamps:** Av = 60 dB, PM = 60°, UGB > 1 GHz; **Novel Opamps:** Av > 100 dB, generations = 800–2000; Running time = 2–9 h
	[48]	Three-stage amplifiers	ATOM	HSpice	FOM = 356.56 × 10^3^, Av = 106.7 dB, UGF = 1.62 MHz, PM = 62.9°, PC = 45.4 μW, generations = 1600, running time ≈ 20 min

**Table 2:** Summary of the efficient parameter optimization methods.

Refs.	Circuit	Optimization Methods	Technology Node	Optimization Results
[51]	Two-stage amplifier	MaxFit GA	180 nm	Av = 76.3 dB, PM = 68.8°, UGF = 11.65 MHz, SR = 9.83 V/μs, PC = 0.166 mW
[52]	Two-stage amplifier	MOGA	180 nm	Av = 75.6dB, PM = 62°, UGF = 16.8MHz, SR = 15.8 V/μs, PC = 220 μW
[53]	Two-stage amplifier	Hybrid PSO	65 nm	Av = 12.1 dB, PM = 104°, UGF = 84.8 MHz, SR = 104 V/μs, PC = 76.1 μW
[17]	Two-stage amplifier	MFO + EA/NSGA-II/BO	180 nm	Av = 36.7 dB, UGF = 20.6 MHz, Iq = 400–800 μA, generations = 50, runtime = 2717 s
[57]	VCO, charge pump, amplifier	BBGP-sDFO	40 nm/180 nm	**VCO**: FoM = 4.083, k_min = 339.37 MHz/V, R_min = 0.998, generations = 452, runtime = 2.8 h; **Charge pump**: FoM = 4.74 (±0.31), diff = 8.103 μA, generations = 452, runtime = 3.2 h; **OTA**: Iq = 10.211 μA, Av= 130.985 dB, UGF = 505.5 MHz, PM = 63.34°, generations = 1390, runtime = 6.8 h
[59]	Folded Cascode amplifier, strong latch comparator	DNN-opt	180 nm	**Folded Cascode amplifier**: Average PC = 0.71 mW, PM > 60°, UGB > 30 MHz, setting time < 30 ns, generations = 132, runtime = 3.3 h **Strong latch comparator**: Average PC= 2.65 μW, set delay < 10 ns, reset delay < 6.5 ns, area < 26 μm2 generations = 330, runtime = 3.9 h
[60]	Two-stage amplifier, three-stage amplifier, LDO	MA-opt	180 nm	**Two-stage amplifier**: PC = 0.608 mW, FoM = −2.93, generations = 200, runtime = 0.83 h **Three-stage amplifier**: PC = 0.129 mW, FoM = −3.57, generations = 200, runtime = 0.86 h **LDO**: Iq = 0.216 mA, FoM = −3.13, generations = 200, runtime = 1.13 h
[61]	Two-stage amplifier	ANN + PSO	65 nm	Av = 20.4 dB, UGF = 100.4 MHz, PM = 62.51°, SR =162.2 V/μs, PC=72.1 μW generations = 100, runtime = 208 s

**Table 3:** Summary of the efficient TL methods.

Refs.	Circuit	Optimization Methods	Transfer Range	Transfer Effect
[70]	Two-stage amplifier	TL with frozen layers	180 nm → 65 nm	**Transferred two-stage amplifier**: PC = 398 μW, Av = 50.3 dB, PM = 51°, GBW = 128 MHz, SR = 2.21 V/μs; Samples can be decreased by 90%
[71]	Inverter, folded Cascode amplifier, ∆Σ DAC	TL + BOAS + MLP	45 nm → 32 nm	**Transferred inverter**: Samples can be decreased by 19×; **Transferred folded Cascode amplifier**: Samples can be decreased by 17×; **Transferred ∆Σ DAC**: Samples can be decreased by 150x;
[74]	Two-stage amplifier, three-stage amplifier, bandgap reference	KATO	180 nm → 40 nm	**Transferred two-stage amplifier**: PC = 254.05 μW, Av = 50.29 dB, PM = 83.72°, GBW = 15.05 MHz; **Transferred three-stage amplifier**: PC = 118.47 μW, Av = 74.41 dB, PM = 71.84°, GBW = 2.65 MHz; **Transferred bandgap reference**: Temperature coefficient (TC) = 9.66 ppm/°C, Iq = 5.42 μA, PSRR = 61.99 dB; Iterations can be decreased by 2.52×
[75]	Two-TIA, three-TIA, voltage amplifier, LDO	GCN-RL	180nm → 250/130/65/45 nm Two-TIA ↔ Three-TIA	250 nm: FoM is increased by 84%; 180 nm: FoM is increased by 98%; 65 nm: FoM is increased by 118%; 45 nm: FoM is increased by 100%; Two-TIA → Three-TIA: FoM is increased by 24%; Three-TIA → Two-TIA: FoM is increased by 3.4%
[76]	CT∆Σ ADC	Graph-guided TL + RL	Fourth-order CIFF ↔ Fourth-order CRFB; Third-order CIFF → Fourth-order CIFF	**Fourth-order CIFF → Fourth-order CRFB**: Simulation efficiency increased 11×, PC = 45.52 mW; **Fourth-order CRFB → Fourth-order CIFF:** Simulation efficiency increased 2.2×, PC = 42.43 mW; **Third-order CIFF → Fourth-order CIFF:** Simulation efficiency increased 1.7×, PC increased 5.6%

**Table 4:** Summary of the optimization design methods considering PVT corners.

Refs.	Circuit	Optimization Methods	Technology Node	Robust Optimization Results
[80]	Two-stage amplifier, Cascode amplifier, LDO	PPAAS	GF180MCU/SKY130	Samples are decreased by 1.6×; Simulation efficiency is increased by 4.1×; PVT deviation is decreased
[82]	Two-stage amplifier	Trust-Region Method+RL	45 nm	Average generations are 36; 9 PVT angle validations are completed in 72.6 iterations; Optimization efficiency is increased by 4×
[83]	Strongarm latch; floating inverter amplifier (FIA); offset-compensation sense amplifier (OCSA)	GLOVA	28 nm	Sample efficiency is increased by 80.5×; Simulation time is decreased by 76.0×; 30 PVT corners can be simulated
[84]	Two-stage amplifier; folded-Cascode amplifier, strongarm latch	RobustAnalog	45 nm/180 nm	**Two-stage amplifier:** Simulation times is decreased by 26×; **Folded-Cascode amplifier:** Simulation times is decreased by 14×; **Strongarm Latch**: Simulation times is decreased by 30×; All circuits pass all PVT corner verification under different random seeds
[85]	Voltage reference circuit	PVT-Transfer	180 nm	FoM is increased by 7.8×; Simulation times are decreased by 60%; Total runtime is 1.8~2.8 h; All PVT conners are verified
[87]	Folded-Cascode amplifier; strongarm latch; BGR; FIA	PVTSizing	28 nm/180 nm	Sample efficiency is increased by 8.8×; Optimization time is decreased by 9.8×; All PVT conners are verified

## Data Availability

No new data were created or analyzed in this study.

## Abbreviations

The following abbreviations are used in this manuscript:

Av	Gain
BAG	Berkeley analog generator
BW	Bandwidth
CTSF	Circuit topology synthesis framework
CIFF	Cascade of integrators feed forward
CRFB	Cascade resonator feedback
CNN	Convolutional neural networks
DAC	Digital to analog converter
DAG	Directed acyclic graph
DDPG	Deep deterministic policy gradient
DL	Deep learning
DLL	Delay locked loop
DNN	Deep neural network
DVAE	Deep variational autoencoder
ES	Evolution strategy
FIA	Floating inverter amplifier
FoM	Figure of merit
GA	Genetic algorithm
GBW	Gain bandwidth
GCN	Graph convolutional network
GNN	Graph neuron network
GP	Gaussian process
IC	Integrated circuit
LDO	Low dropout
LLM	Large language model
MTL	Multi-task learning
MVAR	Multivariate value-at-risk
NSGA II	Non-dominated sorting genetic algorithm II
OCSA	Offset-compensation sense amplifier
PC	Power consumption
PM	Phase margin
PPO	Proximal policy optimization
PSRR	Power supply rejection ratio
PVT	Process, voltage, and temperature
RL	Reinforcement learning
SAR ADC	Successive approximation register analog to digital converter
ΔΣ ADC	Sigma-delta analog to digital converter
SR	Slew rate
TC	Temperature coefficient
TD3	twin delayed deep deterministic policy gradient
TIA	Transimpedance amplifier
TL	Transfer learning
UGB	Unity gain bandwidth
VCO	Voltage controlled oscillator
VGAE	Variational graph autoencoder
